# MTHFR C677T and A1298C polymorphisms in dementia susceptibility: evidence from a meta-analytic investigation with disease-specific and ethnicity-stratified analyses

**DOI:** 10.3389/fnagi.2026.1858837

**Published:** 2026-08-12

**Authors:** Yuyao He, Yandan Shi, Jinhua Hu, Xiyuan Wang, Jinqing Su, Ziyuan Yang, Zhihao Zhang, Sha Liao, Xiuxian Lu, Xiqi Zhu

**Affiliations:** 1 School of Medical Technology and Artificial Intelligence, Youjiang Medical university for Nationalities, Baise, China; 2 Department of Radiology, Affiliated Hospital of Youjiang Medical university for Nationalities, Baise, China; 3 Guangxi Engineering Research Center for Precise Genetic Testing of Long-dwelling Nationalities, Guangxi, China

**Keywords:** Alzheimer’s disease, dementia, genetic polymorphism, methylenetetrahydrofolate reductase, vascular dementia

## Abstract

**Background:**

Alzheimer’s disease (AD) and vascular dementia (VaD) have become significant global health challenges. Recent research evidence indicates a close comorbidity between AD and cerebral small vessel disease. The C677T (rs1801133, also known as c.665C > T) and A1298C (rs1801131) polymorphisms in the methylenetetrahydrofolate reductase (*MTHFR*) gene have been associated with both conditions, though conclusions vary across studies.

**Methods:**

We conducted a systematic search of PubMed, Embase, Web of Science and the Cochrane Library, covering the period from the inception of these databases to January 2026. Studies reporting the distribution of *MTHFR* C677T and/or A1298C genotypes were included. A fixed-effects model was used to calculate the pooled odds ratio (OR) and its 95% confidence interval (CI). Sensitivity analyses using random-effects models were also performed to test the robustness of the findings. Subgroup analyses were performed according to type of dementia and ethnicity.

**Results:**

A total of 26 C677T studies and 8 A1298C studies were included. In the allele contrast model (T vs. C), C677T was significantly associated with dementia (OR = 1.40, 95% CI 1.05–1.87, *p* = 0.021) and AD (OR = 1.40, 95% CI 1.01–1.93, *p* = 0.043), but no statistically significant association was observed for VaD (OR = 1.42, 95% CI 0.76–2.66, *p* = 0.266). C677T showed no significant association under homozygous, heterozygous, dominant, or recessive inheritance models. No significant heterogeneity in association was observed across racial subgroups. A1298C showed no significant association with dementia under any genetic model.

**Conclusion:**

This meta-analysis showed that the *MTHFR* C677T T allele is associated with the risk of AD in an allele-dependent model, with a similar but non-significant trend for VaD. The A1298C polymorphism shows no association with either type of dementia. These findings suggest that C677T genotyping may aid AD risk stratification in research, but it requires prospective validation and gene-environment studies before clinical use.

## 1 Introduction

Dementia, particularly Alzheimer’s disease (AD) and vascular dementia (VaD), represents a significant global health challenge, with its pathogenesis involving the interaction of multiple factors including genetics and environment. Recent research indicates a co-morbid mechanism between AD and cerebral small vessel disease (CSVD), suggesting potential bidirectional influences between the two conditions during disease progression. The folate metabolic pathway participates in homocysteine regulation and the maintenance of neurovascular homeostasis; abnormalities in this pathway are closely associated with both neurodegeneration and vascular injury (Quadri et al., 2004; Seshadri et al., 2002). Methylenetetrahydrofolate reductase (*MTHFR*), a core enzyme in this pathway, exhibits genetic polymorphisms that may influence AD and VaD pathogenesis by regulating homocysteine levels (Frosst et al., 1995; Rozen, 1997). Whether *MTHFR* polymorphisms exert differential effects on AD versus VaD remains unclear. Addressing this question is essential for understanding subtype-specific genetic architecture.

The *MTHFR* gene harbors two common functional single nucleotide polymorphisms: C677T (rs1801133, also known as c.665C > T) and A1298C (rs1801131). The C677T variant has been definitively demonstrated to reduce enzyme activity, leading to elevated homocysteine levels (Devlin et al., 2006; Frosst et al., 1995; Kluijtmans et al., 2003). This may increase dementia risk through pathways involving vascular damage, oxidative stress, and neurotoxicity (Kruman et al., 2002; Mattson and Shea, 2003). In contrast, findings regarding the impact of the A1298C variant on enzyme activity and its association with dementia are inconsistent and often contradictory. Whether it independently correlates with dementia risk separate from C677T remains unclear. Therefore, this study will further investigate whether A1298C is associated with dementia risk.

Despite extensive research examining the association between *MTHFR* C677T and A1298C polymorphisms and dementia risk, the findings remain inconsistent (Brunelli et al., 2001; Chapman et al., 1998; Linneba et al., 2004; Zuliani et al., 2001a). This discrepancy may stem from: (i) the use of different genetic models, which can influence the sensitivity of association detection; (ii) variations in allele frequencies across ethnic populations, which may lead to differing observed effect sizes (Pepe et al., 1998; Rai et al., 2012; Schneider et al., 1998; Wilcken et al., 2003). To further resolve these controversies, it is necessary to conduct a comparative analysis stratified by genetic model and ethnicity. This systematic investigation aims to determine whether differences exist in the sensitivity of detecting this association across various genetic models, and whether the link between *MTHFR* polymorphisms and dementia risk exhibits racial universality.

To resolve the aforementioned controversy, this study conducted a comprehensive, stratified meta-analysis. The aim was to systematically evaluate the association between *MTHFR* C677T and A1298C polymorphisms and the risk of dementia (as a general diagnostic category, encompassing AD and VaD), as well as AD and VaD separately. The core objectives of this study include: (i) determining whether the C677T polymorphism is primarily associated with specific dementia subtypes; (ii) comparing the detection efficacy of different genetic models; (iii) validating the universality of this association across different ethnic populations; and (iv) ultimately clarifying the role of the A1298C polymorphism in dementia risk.

## 2 Materials and methods

### 2.1 Protocol and registration

This meta-analysis was conducted in strict adherence to the Preferred Reporting Items for Systematic Reviews and Meta-Analyses (PRISMA) statement, 2020 edition (PRISMA-2020). The study protocol has been prospectively registered in the International Prospective Registration Database for Systematic Reviews (registration number: CRD420261306825).

### 2.2 Search strategy and selection criteria

The databases PubMed, Embase, Web of Science, and the Cochrane Library were systematically searched from inception to January 2026, supplemented by other sources. The search strategy combined subject headings and free-text terms related to “*MTHFR*,” “C677T” (or “C.665C > T”), “A1298C,” “polymorphism,” “Alzheimer’s disease,” and “vascular dementia,” along with the rs numbers (rs1801133 for C677T and rs1801131 for A1298C) to maximize sensitivity and reduce nomenclature-related selection bias. Boolean operators were used to integrate the terms.

Inclusion criteria: (i) The case group comprises patients with AD or VaD meeting recognized diagnostic criteria (NINCDS-ADRDA, NINDS-AIREN, DSM); the control group comprises individuals with normal cognitive function or assessed as free of dementia symptoms. (ii) Genotype frequencies at the *MTHFR* C677T and/or A1298C loci must be determined and reported. (iii) Complete data enabling calculation of OR and 95% CI must be provided. (iv) Only articles published in full in English are eligible.

Exclusion criteria: (i) Literature types such as reviews, meta-analyses, animal studies, case reports, and letters to the editor; (ii) Studies where full-text or complete genotype data were unavailable; (iii) Studies involving adolescents, individuals with concomitant severe neurological disorders, or those with unclear cognitive impairment.

### 2.3 Data extraction and quality assessment

Two researchers independently extracted the following information: first author, publication year, country, ethnicity, sample size, dementia type, genotyping method, genetic locus, genotype distribution in case and control groups and *p*-value of HWE (either as reported in the original studies or recalculated from the available genotype frequency data). In this study, “ethnicity” was categorized based on geographical and genetic background, standardized into East Asian populations, European populations, Middle Eastern and West Asian populations, South Asian populations, African and Mixed-Race populations.

Given the specific requirements of genetic association studies, this study assessed methodological quality using both the Newcastle-Ottawa Scale (NOS) and the Q-Genie tool. The NOS evaluates studies across three dimensions: selection of study subjects (maximum score of 4), comparability between groups (maximum score of 2), and assessment of exposure/outcome (maximum score of 3), with a total score ranging from 0 to 9; studies scoring ≥ 7 are classified as high-quality. The Q-Genie tool assesses the quality of genetic association studies across 11 dimensions, including genotyping quality control, Hardy-Weinberg equilibrium, control for population stratification, genetic model specification, and correction for multiple testing, with each item scored from 1 to 7 points, resulting in a total score ranging from 11 to 77 points. According to the Q-Genie scoring criteria, for studies with a control group, a total score > 45 indicates good quality; for studies without a control group, a total score > 40 indicates good quality. All included studies were comprehensively assessed based on the above criteria.

### 2.4 Statistical analysis

The OR and its 95% CI were employed to assess the strength of association between *MTHFR* polymorphisms and dementia risk. For the C677T locus, the following genetic models were analyzed: allele contrast model (T vs. C), homozygous model (TT vs. CC), heterozygous/codominant model (CT vs. CC), recessive model (TT vs. TC + CC), and dominant model (TT + CT vs. CC). For the A1298C locus, the following genetic models were analyzed: allele contrast model (C vs. A), homozygous model (CC vs. AA), heterozygous/codominant model (AC vs. AA), dominant model (AC + CC vs. AA), and recessive model (CC vs. AA + AC).

Heterogeneity among studies was assessed using the I^2^ statistic and *p*-values. As no significant heterogeneity was observed in the analyses (*I*^2^ = 0%, *p* > 0.10), a fixed effects model was adopted as the primary analytical approach; to test the robustness of the results (regardless of whether the assumption of homogeneity was met), we also conducted analyses using a random effects model for all pooled estimates. Subgroup analyses were conducted by dementia type and ethnicity. All statistical analyses were performed using Stata 16.0.

### 2.5 Publication bias

Funnel plots, Begg’s test, and Egger’s test were employed to assess potential publication bias, with *p* > 0.05 indicating no significant bias.

## 3 Results

### 3.1 Literature search and study selection

**FIGURE 1:**
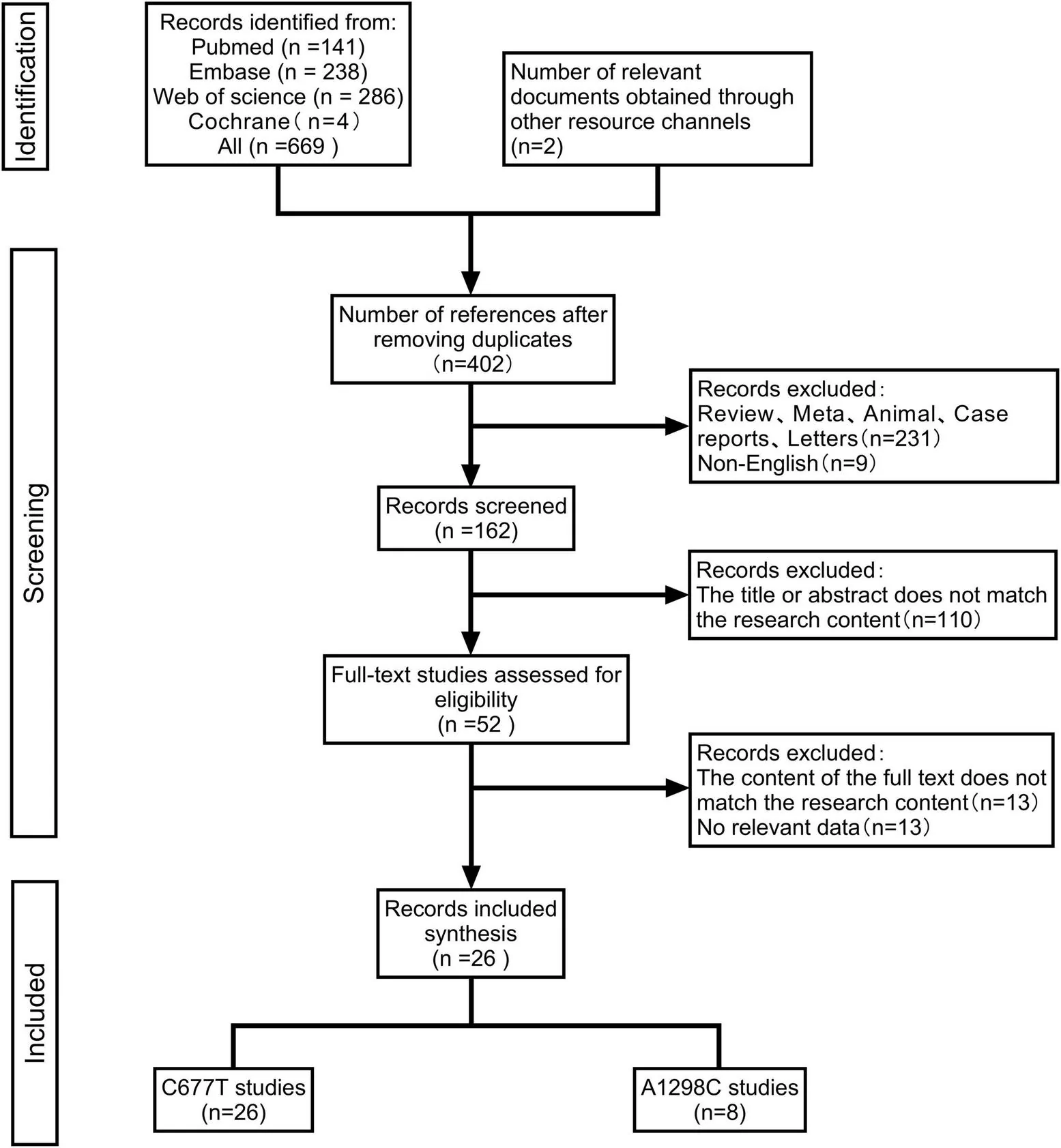
Flow diagram of selection of studies included in the meta-analysis.

A total of 671 relevant publications were retrieved. After removing duplicates, 402 remained. Following preliminary screening, 350 publications were excluded: reviews, meta-analyses, animal studies, case reports, letters to the editor, non-English language publications, and those irrelevant to the topic. Following a full-text assessment of the remaining 52 documents, a further 26 were excluded: 13 due to irrelevance and 13 due to inaccessibility. Ultimately, a total of 26 papers were included in the meta-analysis. All 26 studies reported data on the *MTHFR* C677T polymorphism, and eight of them also provided data on the A1298C polymorphism (Figure 1).

### 3.2 Study characteristics

The baseline characteristics included in the study are presented in Table 1. In the *MTHFR* C677T analysis, the proportions of CC, CT, and TT genotypes in the case group were 41.22, 42.59, and 16.19%, respectively; in the control group, these proportions were 48.76, 38.71, and 12.52% (Supplementary Table 1). In the A1298C analysis, the proportions of AA, AC, and CC genotypes in the case group were 56.18, 34.92, and 8.90%, respectively; in the control group, these proportions were 47.45, 35.07, and 17.49% (Supplementary Table 2).

**TABLE 1:** Details of studies included in the present meta-analysis.

Study	Country	Ethnicity	Types of dementia	Gene locus	Case samples	Control samples	*P*-value of HWE ^c^
Belcavello et al. (2015)	Brazil	African and Mixed-Race populations	AD ^a^	C677T	82	161	> 0.05
Bi et al. (2009)	China	East Asian populations	AD ^a^	C677T	386	375	> 0.05
Keikhaee et al. (2006)	Iran	Middle Eastern and West Asian populations	AD ^a^	C677T	117	125	> 0.05
Rizi et al. (2024)	Iran	Middle Eastern and West Asian populations	AD ^a^	C677T	88	100	> 0.05
Nishiyama et al. (2000)	Japan	East Asian populations	AD ^a^	C677T	24	33	0.845
Wehr et al. (2006)	Poland	European populations	AD ^a^	C677T	99	141	> 0.05
Religa et al. (2003)	Poland	European populations	AD ^a^	C677T	99	100	0.901
Li et al. (2009)	China	East Asian populations	AD ^a^	C677T	198	240	> 0.05
Chhillar et al. (2014)	India	South Asian populations	AD ^a^	C677T	100	100	> 0.05
Fernandez and Scheibe (2005)	Brazil	European populations	AD ^a^	C677T	30	29	0.648
Seripa et al. (2003)	United States	European populations	AD ^a^	C677T	124	97	> 0.05
Seripa et al. (2003)	Italy	European populations	AD ^a^	C677T	126	106	> 0.05
Bouguerra et al. (2024)	Algeria	African and Mixed-Race populations	AD ^a^	C677T	106	104	> 0.05
Mansoori et al. (2012)	India	South Asian populations	AD ^a^	C677T	80	120	0.835
Hamad et al. (2024)	Sudan, Oman, Saudi Arabia	African and Mixed-Race populations	AD ^a^	C677T	73	73	> 0.05
Elhawary et al. (2013)	Egypt, Saudi Arabia	African and Mixed-Race populations	AD ^a^	C677T	43	32	0.630
Pollak et al. (2000)	Israel	Middle Eastern and West Asian populations	AD ^a^	C677T	92	82	> 0.05
da Silva et al. (2006)	Brazil	African and Mixed-Race populations	AD ^a^	C677T	43	50	> 0.05
Styczyńska et al. (2008)	Poland	European populations	AD ^a^	C677T	154	176	0.924
Mansouri et al. (2013)	Tunisia	African and Mixed-Race populations	AD ^a^	C677T	38	100	0.667
Jiang et al. (2021)	China	East Asian populations	AD ^a^	C677T	721	365	> 0.05
McIlroy et al. (2002)	United Kingdom/ Northern Ireland	European populations	AD ^a^	C677T	83	71	0.902
Jin et al. (2013)	China	East Asian populations	VaD ^b^	C677T	304	300	> 0.05
Kaur et al. (2018)	India	South Asian populations	VaD ^b^	C677T	234	298	0.525
Zuliani et al. (2001a)	Italy	European populations	VaD ^b^	C677T	60	54	0.335
Nishiyama et al. (2000)	Japan	East Asian populations	VaD ^b^	C677T	35	33	0.866
Chen et al. (2023)	China	East Asian populations	VaD ^b^	C677T	191	182	> 0.05
Mansoori et al. (2012)	India	South Asian populations	VaD ^b^	C677T	50	120	0.768
Pollak et al. (2000)	Israel	Middle Eastern and West Asian populations	VaD ^b^	C677T	85	82	> 0.05
Zuliani et al. (2001b)	Italy	European populations	VaD ^b^	C677T	68	20	0.104
McIlroy et al. (2002)	United Kingdom/ Northern Ireland	European populations	VaD ^b^	C677T	78	71	0.452
Bouguerra et al. (2024)	Algeria	African and Mixed-Race populations	AD ^a^	A1298C	102	51	> 0.05
Mansoori et al. (2012)	India	South Asian populations	AD ^a^	A1298C	80	120	0.696
Hamad et al. (2024)	Sudan, Oman, Saudi Arabia	African and Mixed-Race populations	AD ^a^	A1298C	73	73	> 0.05
da Silva et al. (2006)	Brazil	African and Mixed-Race populations	AD ^a^	A1298C	43	50	> 0.05
Mansouri et al. (2013)	Tunisia	African and Mixed-Race populations	AD ^a^	A1298C	38	100	0.720
Jiang et al. (2021)	China	East Asian populations	AD ^a^	A1298C	721	365	> 0.05
Chen et al. (2023)	China	East Asian populations	VaD ^b^	A1298C	191	182	> 0.05
Mansoori et al. (2012)	India	South Asian populations	VaD ^b^	A1298C	50	120	0.340

^a^AD, Alzheimer’s disease.

^b^VaD, vascular dementia.

^c^HWE, Hardy-Weinberg Equilibrium. The HWE *p*-values for the control group were either taken directly from the original paper or recalculated based on the reported genotype frequencies where data were available.

### 3.3 Quality assessment

The NOS was employed to assess the methodological quality of the 26 included studies. Results indicated that 24 studies demonstrated high quality (NOS ≥ 7), while 2 studies were rated as moderate quality (NOS = 6), yielding an overall good quality of evidence (Supplementary Table 3). According to the Q-Genie criteria, all 26 studies were rated as good quality (Supplementary Table 4).

### 3.4 Specific association between *MTHFR* C677T polymorphism and dementia and its subtypes

**FIGURE 2:**
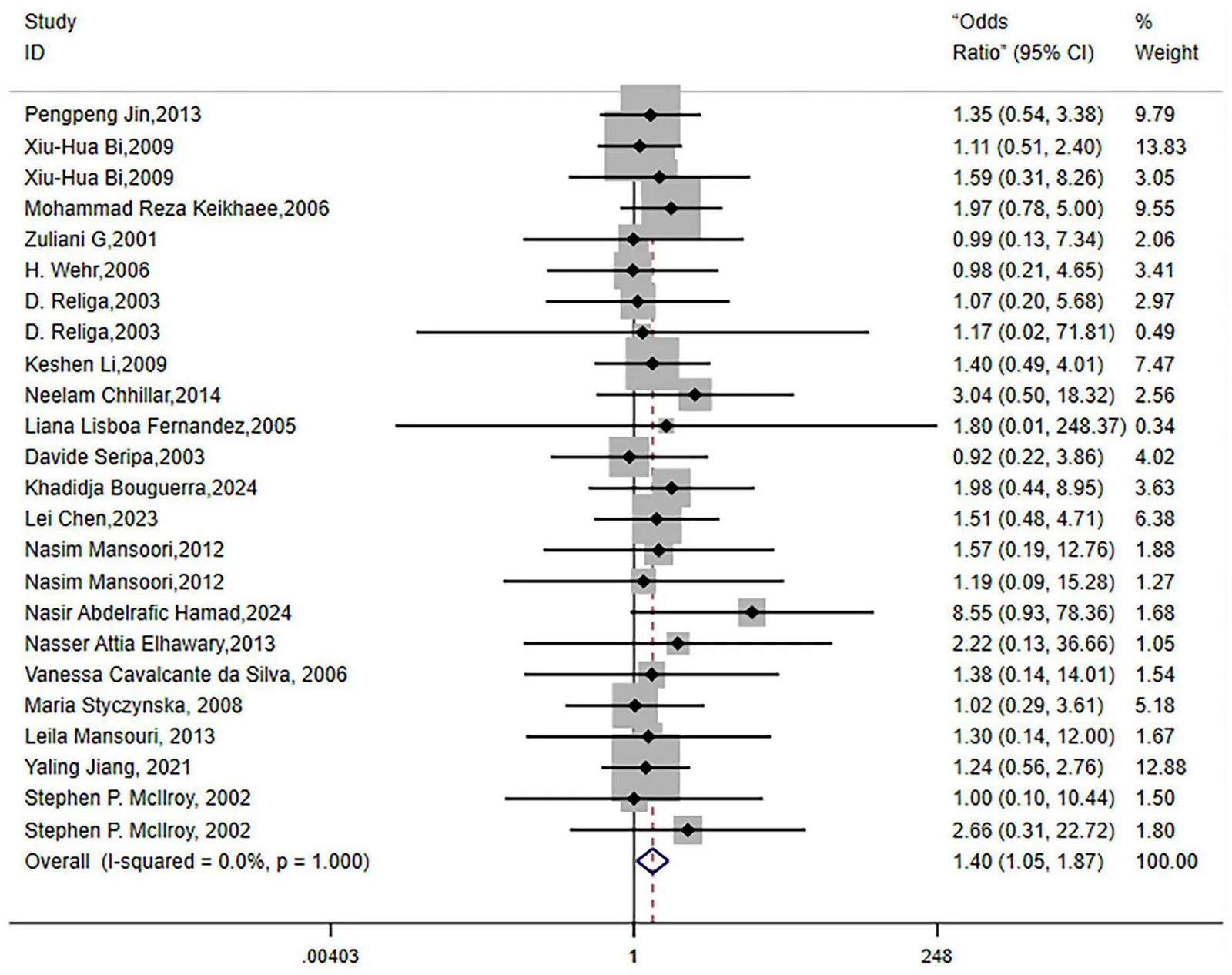
Forest plot of the association between *MTHFR* C677T polymorphism and dementia risk (allele contrast model T vs. C).

**FIGURE 3:**
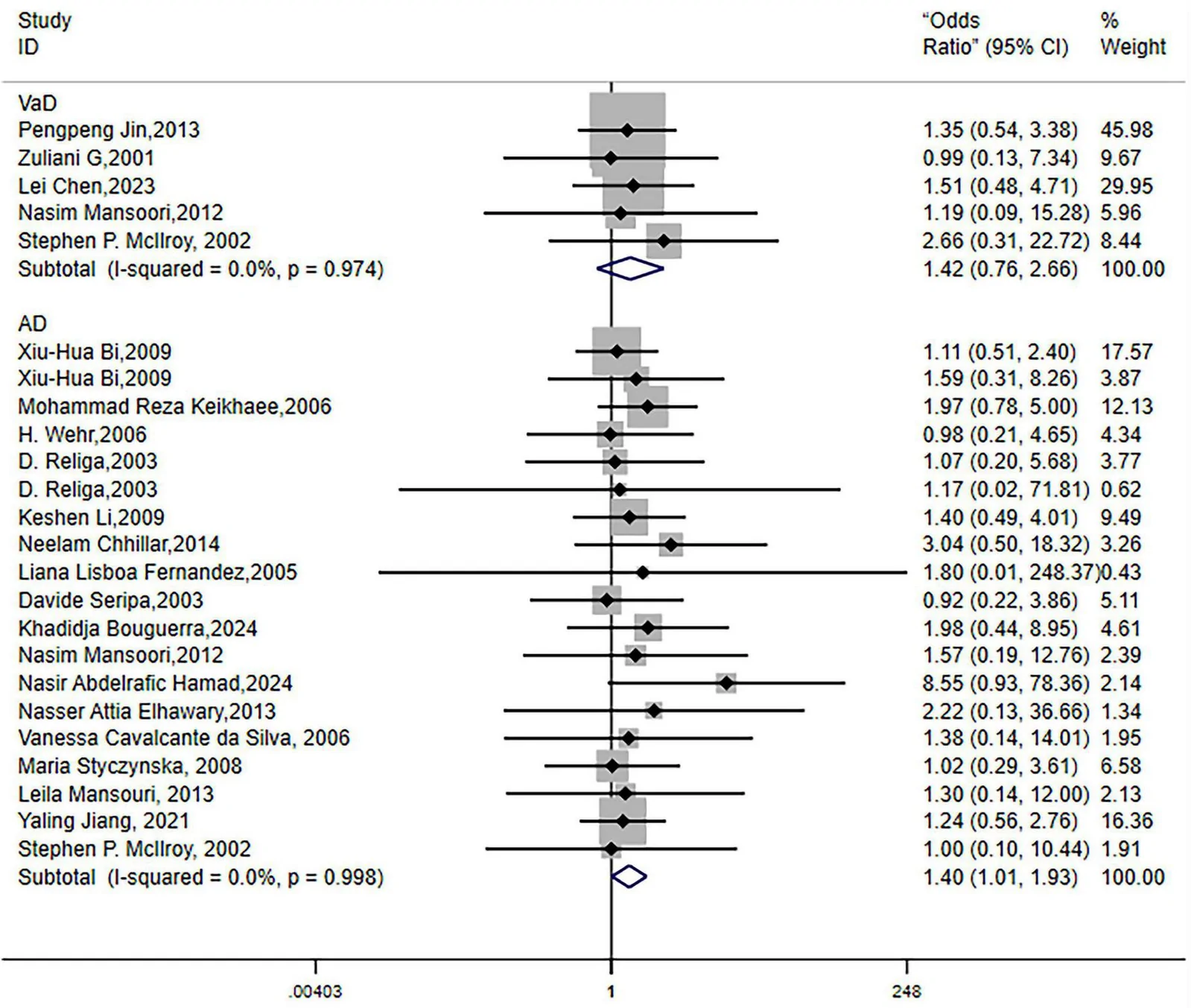
Subgroup forest plot by dementia subtype (AD vs. VaD) for the *MTHFR* C677T polymorphism under the allele contrast model (T vs. C).

A meta-analysis of the *MTHFR* C677T allele contrast model (T vs. C) revealed a significant association between the T allele and dementia risk (OR = 1.40, 95% CI = 1.05–1.87, *p* = 0.021) (Figure 2 and Table 2). Subgroup analysis further revealed that this association was primarily observed in the AD subgroup (OR = 1.40, 95% CI = 1.01–1.93, *p* = 0.043) (Belcavello et al., 2015; Bi et al., 2009; Keikhaee et al., 2006; Rizi et al., 2024; Nishiyama et al., 2000; Wehr et al., 2006; Religa et al., 2003; Li et al., 2009; Chhillar et al., 2014; Fernandez and Scheibe, 2005; Seripa et al., 2003; Bouguerra et al., 2024; Mansoori et al., 2012; Hamad et al., 2024; Elhawary et al., 2013; Pollak et al., 2000; da Silva et al., 2006; Styczyńska et al., 2008; Mansouri et al., 2013; Jiang et al., 2021; McIlroy et al., 2002). In contrast, no significant association was observed in the VaD subgroup (OR = 1.42, 95% CI = 0.76–2.66, *p* = 0.266) (Figure 3 and Table 2; Jin et al., 2013; Kaur et al., 2018; Zuliani et al., 2001a; Nishiyama et al., 2000; Chen et al., 2023; Mansoori et al., 2012; Pollak et al., 2000; Zuliani et al., 2001b; McIlroy et al., 2002). This suggests a potential disease specific effect of the *MTHFR* C677T variant: a significant association was observed for AD, while the effect for VaD pointed in the same direction but was not statistically significant, warranting cautious interpretation due to the limited number of VaD studies and wide confidence intervals.

**TABLE 2:** Summary meta-analysis results for the *MTHFR* C677T polymorphism.

Genetic models	Fixed effect OR ^a^ (95 % CI ^b^), *p*	Random effect OR ^a^ (95 % CI ^b^), *p*	Heterogeneity *p*-value (Q test)	I^2^ (%)	Publication bias (p of Begg’s test)	Publication bias (p of Egger’s test)
Total studies						
midrule Allele contrast (T vs. C)	1.40(1.05, 1.87), 0.021	1.40(1.05, 1.87), 0.021	1.000	0	0.206	0.244
Homozygote (TT vs. CC)	1.53(0.70, 3.33), 0.284	1.53(0.70, 3.33), 0.284	1.000	0	0.495	0.880
Heterozygote(CT vs. CC)	1.38(0.73, 2.63), 0.321	1.38(0.73, 2.63), 0.321	0.996	0	0.381	0.338
Dominant (TT + CT vs. CC)	1.30(0.76, 2.23), 0.343	1.30(0.76, 2.23), 0.343	0.990	0	0.096	0.109
Recessive (TT vs. CT + CC)	1.48(0.56, 3.96), 0.431	1.48(0.56, 3.96), 0.431	1.000	0	0.583	0.271
AD						
Allele contrast (T vs. C)	1.40(1.01, 1.93), 0.043	1.40(1.01, 1.93), 0.043	0.998	0	0.208	0.266
Homozygote (TT vs. CC)	1.54(0.67, 3.54), 0.314	1.54(0.67, 3.54), 0.314	1.000	0	0.661	0.747
Heterozygote(CT vs. CC)	1.46(0.72, 2.94), 0.29	1.46(0.72, 2.94), 0.29	0.992	0	0.213	0.177
Dominant (TT + CT vs. CC)	1.48(0.78, 2.82), 0.233	1.48(0.78, 2.82), 0.233	0.980	0	0.200	0.364
Recessive (TT vs. CT + CC)	1.33(0.48, 3.73), 0.583	1.33(0.48, 3.73), 0.583	1.000	0	0.721	0.950
VaD						
Allele contrast (T vs. C)	1.42(0.76, 2.66), 0.266	1.42(0.76, 2.66), 0.266	0.974	0	0.806	0.860
Homozygote (TT vs. CC)	1.50(0.18, 12.68), 0.711	1.50(0.18, 12.68), 0.711	0.953	0	1	0.173
Heterozygote(CT vs. CC)	1.05(0.22, 5.13), 0.951	1.05(0.22, 5.13), 0.951	0.664	0	1	0.522
Dominant (TT + CT vs. CC)	0.95(0.35, 2.57), 0.922	0.95(0.35, 2.57), 0.922	0.786	0	1	0.369
Recessive (TT vs. CT + CC)	4.51(0.16, 124.93), 0.374	4.51(0.16, 124.93), 0.374	0.998	0	1	0.709
East Asian populations						
Allele contrast (T vs. C)	1.29(0.87, 1.92), 0.201	1.29(0.87, 1.92), 0.201	0.997	0	0.024	0.03
Homozygote (TT vs. CC)	1.49(0.49, 4.50), 0.483	1.49(0.49, 4.50), 0.483	0.919	0	1	0.297
Heterozygote(CT vs. CC)	1.28(0.44, 3.68), 0.684	1.28(0.44, 3.68), 0.684	0.949	0	1	0.860
Dominant (TT + CT vs. CC)	0.84(0.26, 2.71), 0.771	0.84(0.26, 2.71), 0.771	0.827	0	0.296	0.051
Recessive (TT vs. CT + CC)	2.68(0.42, 17.30), 0.299	2.68(0.42, 17.30), 0.299	0.967	0	1	0.163
European populations						
Allele contrast (T vs. C)	1.09(0.59, 2.02), 0.780	1.09(0.59, 2.02), 0.780	1.000	0	0.133	0.059
Homozygote (TT vs. CC)	1.28(0.24, 6.70), 0.773	1.28(0.24, 6.70), 0.773	0.997	0	0.806	0.105
Heterozygote(CT vs. CC)	1.11(0.32, 3.89), 0.871	1.11(0.32, 3.89), 0.871	0.941	0	0.734	0.596
Dominant (TT + CT vs. CC)	1.25(0.56, 2.81), 0.581	1.25(0.56, 2.81), 0.581	0.997	0	0.536	0.091
Recessive (TT vs. CT + CC)	1.18(0.36, 3.87), 0.785	1.18(0.36, 3.87), 0.785	1.000	0	0.063	0.018
South Asian populations						
Allele contrast (T vs. C)	1.99(0.60, 6.61), 0.264	1.99(0.60, 6.61), 0.264	0.811	0	0.296	0.222
Heterozygote(CT vs. CC)	1.69(0.26, 11.05), 0.582	1.69(0.26, 11.05), 0.582	0.185	43.1	1	/
Dominant (TT + CT vs. CC)	1.75(0.28, 11.05), 0.552	1.75(0.28, 11.05), 0.552	0.181	44.1	1	/
African and Mixed-Race populations						
Allele contrast (T vs. C)	2.27(0.90, 5.76), 0.083	2.27(0.90, 5.76), 0.083	0.768	0	1	0.880
Homozygote (TT vs. CC)	2.56(0.39, 16.97), 0.331	2.56(0.39, 16.97), 0.331	0.869	0	0.806	0.791
Heterozygote(CT vs. CC)	1.77(0.50, 6.30), 0.378	1.77(0.50, 6.30), 0.378	0.937	0	0.462	0.160
Dominant (TT + CT vs. CC)	2.89(0.50, 2.81), 0.234	2.89(0.50, 2.81), 0.234	0.997	0	1	0.113
Recessive (TT vs. CT + CC)	1.20(0.01, 190.23), 0.945	1.20(0.01, 190.23), 0.945	0.650	0	1	/
Middle Eastern and West Asian populations						
Homozygote (TT vs. CC)	0.71(0.05, 9.71), 0.975	0.71(0.05, 9.71), 0.975	1.000	0	0.734	0.466
Dominant (TT + CT vs. CC)	1.35(0.34, 5.27), 0.975	1.35(0.34, 5.27), 0.975	0.372	0	1	/

^a^ Odds ratios.

^b^ Confidence intervals.

### 3.5 Comparison of the performance of different genetic models in detecting the association of *MTHFR* C677T

Beyond the allele model, this study evaluated the association between the C677T polymorphism and the risk of dementia, AD, and VaD across multiple genetic models (Table 2 and Supplementary Figures 1, 2). Results indicated: the homozygous model (TT vs. CC) showed no significant associated with dementia (OR = 1.53, 95% CI = 0.70–3.33, *p* = 0.284), AD (OR = 1.54, 95% CI = 0.67–3.54, *p* = 0.314) or VaD (OR = 1.50, 95% CI = 0.18–12.68, *p* = 0.711); the heterozygous model (CT vs. CC) showed no significant association with dementia (OR = 1.38, 95% CI = 0.73–2.63, *p* = 0.321), AD (OR = 1.46, 95% CI = 0.72–2.94, *p* = 0.29) or VaD (OR = 1.05, 95% CI = 0.22–5.13, *p* = 0.951); the dominant model (TT+CT vs. CC) showed no significant association with dementia (OR = 1.30, 95% CI = 0.76–2.23, *p* = 0.343), AD (OR = 1.48, 95% CI = 0.78–2.82, *p* = 0.233) or VaD (OR = 0.95, 95% CI = 0.35–2.57, *p* = 0.922). The recessive model (TT vs. CC+CT) showed no significant association with dementia (OR = 1.48, 95% CI = 0.56–3.96, *p* = 0.431), AD (OR = 1.33, 95% CI = 0.48–3.73, *p* = 0.583) or VaD (OR = 4.51, 95% CI = 0.16–124.93, *p* = 0.374).

The allele model (T vs. C) demonstrated higher sensitivity in detecting the association between the C677T polymorphism and dementia (particularly AD), whereas no significant association was detected under homozygous, heterozygous, dominant, or recessive models (Belcavello et al., 2015; Bi et al., 2009; Chen et al., 2023; Chhillar et al., 2014; da Silva et al., 2006; Jin et al., 2013; Keikhaee et al., 2006; Li et al., 2009; Mansouri et al., 2013; McIlroy et al., 2002; Pollak et al., 2000; Rizi et al., 2024; Seripa et al., 2003; Styczyńska et al., 2008; Wehr et al., 2006).

### 3.6 Consistent effect of *MTHFR* C677T on dementia across ethnic groups

Subgroup analyses by ethnicity revealed (Table 2 and Supplementary Figure 3) no significant differences in the strength of association between the C677T T allele and dementia risk across different ethnic subgroups. Results from all genotyping models showed consistent trends (all 95% confidence intervals for odds ratios included 1, and all *P*-values were > 0.05). This indicates that, despite potential variations in allele frequencies across racial groups, the association between the C677T polymorphism and dementia risk (particularly AD) remains consistent across populations with differing genetic backgrounds, with no significant racial specificity observed (da Silva et al., 2006; McIlroy et al., 2002; Pollak et al., 2000; Religa et al., 2003; Styczyńska et al., 2008; Zuliani et al., 2001a; Zuliani et al., 2001b).

### 3.7 Assessment of the role of the *MTHFR* A1298C polymorphism in dementia risk

For the *MTHFR* A1298C polymorphism, no significant association with dementia risk was observed across all genetic models (Table 3 and Supplementary Figure 4; Bouguerra et al., 2024; Chen et al., 2023; Mansoori et al., 2012; Hamad et al., 2024; da Silva et al., 2006; Jiang et al., 2021; Mansouri et al., 2013): Allele contrast model (C vs. A) (OR = 1.18, 95% CI = 0.67–2.09, *p* = 0.596), homozygous model (CC vs. AA) (OR = 1.19, 95% CI = 0.02–70.37, *p* = 0.935), heterozygous/codominant model (AC vs. AA) (OR = 1.94, 95% CI = 0.31–12.08, *p* = 0.479), dominant model (AC + CC vs. AA) (OR = 2.54, 95% CI = 0.47–13.82, *p* = 0.28), and recessive model (CC vs. AA+AC) (OR = 13.92, 95% CI = 0.27–706.88, *p* = 0.198) all yielded negative results in dementia analysis. These consistent negative findings suggest that the A1298C polymorphism may not play a primary or independent role in dementia risk pathways.

**TABLE 3:** Summary meta-analysis results for the *MTHFR* A1298C polymorphism.

Genetic models	Fixed effect OR ^a^ (95 % CI ^b^), *p*	Random effect OR ^a^ (95 % CI ^b^), *p*	Heterogeneity *p*-value (Q test)	I^2^ (%)	Publication bias (p of Begg’s test)	Publication bias (p of Egger’s test)
Total studies						
Allele contrast (C vs. A)	1.18(0.67, 2.09), 0.569	1.18(0.67, 2.09), 0.569	0.961	0	0.174	0.227
Homozygote (CC vs. AA)	1.19(0.02, 70.37), 0.935	1.19(0.02, 70.37), 0.935	0.987	0	1	/
Heterozygote(AC vs. AA)	1.94(0.31, 12.08), 0.479	1.94(0.31, 12.08), 0.479	0.399	0	0.296	0.187
Dominant (AC + CC vs. AA)	2.54(0.47, 13.82), 0.280	2.54(0.47, 13.82), 0.280	0.494	0	1	0.484
Recessive (CC vs. AA + AC)	13.92(0.27, 706.88), 0.198	13.92(0.27, 706.88), 0.198	0.636	0	1	/

^a^Odds ratios.

^b^Confidence intervals.

### 3.8 Publication bias

Publication bias in the included studies was assessed using funnel plots in conjunction with Begg’s and Egger’s tests. For the C677T polymorphism (26 studies), the funnel plot exhibited a nearly symmetrical shape with no obvious skewed distribution, and the *p*-values for both Begg’s and Egger’s tests were greater than 0.05, indicating no significant evidence of publication bias. For the A1298C polymorphism (8 studies), the aforementioned tests similarly failed to reveal statistical significance (all *p* > 0.05); however, given the limited number of studies (*n* = 8), the statistical power of these tests to detect publication bias is inherently low, and these results should therefore be interpreted with caution.

### 3.9 Sensitivity analysis

To assess whether the choice of meta-analytic model influenced the conclusions, we re-analyzed all pooled estimates using random effects models. The direction, magnitude and statistical significance of the effect sizes remained essentially unchanged compared with the fixed effects models (Tables 2, 3). This indicates that the overall findings are robust to the choice of statistical model.

## 4 Discussion

This stratified meta-analysis aimed to clarify the disease-specific roles of *MTHFR* polymorphisms in AD and VaD. Four main findings emerged. First, The *MTHFR* C677T T allele is a significant risk factor for dementia, particularly AD. A similar directional effect was observed for VaD, but it did not reach statistical significance. Second, a significant association was observed only in the allele model, not in other genetic models. Third, no significant differences in this risk effect were observed across racial groups. Fourth, the *MTHFR* A1298C polymorphism showed no significant association with the risk of either AD or VaD.

This study found that C677T is associated solely with AD risk and not with VaD. A prior meta-analysis by Zhang et al. (2010), conducted a meta-analysis employing an allele-comparison model, similarly reported a significant association between the C677T polymorphism and AD risk. However, their analysis did not perform a direct comparison between AD and VaD subgroups, leaving the question of disease specificity unresolved. The absence of association with VaD in our analysis may seem counterintuitive. While hyperhomocysteinemia is a well-established risk factor for vascular disease (Seshadri et al., 2002), its pathogenic role may differ between dementia subtypes. Experimental studies have shown that homocysteine can exacerbate AD-specific pathology by impairing DNA repair in hippocampal neurons and sensitizing them to β-amyloid toxicity (Kruman et al., 2002), as well as by inducing intraneuronal accumulation of neurotoxic Aβ42 (Hasegawa et al., 2005). These mechanisms are directly relevant to AD neuropathology. Furthermore, elevated homocysteine levels have been associated with accelerated brain atrophy, which may contribute to cognitive decline in neurodegenerative diseases (Sachdev, 2005; Stühlinger et al., 2003). In contrast, while homocysteine undoubtedly contributes to endothelial dysfunction and thrombogenesis (Kartal Ozer et al., 2005; Midorikawa et al., 2000; Stühlinger et al., 2003), and although the thermolabile *MTHFR* variant has been directly implicated in VaD pathogenesis (Yoo et al., 2000), these vascular effects may require additional co-factors to culminate in clinically manifest VaD, such as hypertension, diabetes, or advanced atherosclerosis. Furthermore, the current number of studies and sample sizes included in the VaD subgroup are relatively limited, potentially leading to insufficient statistical power. This may explain why no significant associations were detected. Future validation requires larger-scale studies.

A notable finding of this study is that the significant association between C677T and dementia was detected only under the allele-comparison model, whereas no significant association was observed under homozygous, heterozygous, dominant, or recessive models. This pattern warrants cautious interpretation and differs from the results of some previous meta-analyses. Our findings partially align with the meta-analysis by Rai (2017), which also reported a significant association under the allele model. However, Rai’s analysis identified significant associations under both dominant and recessive models, whereas this study did not observe such effects. This discrepancy may reflect differences in study inclusion criteria and sample composition. This study failed to identify significant associations under the non-allelic genetic models, suggesting that the effect size of the C677T polymorphism may be small. Larger sample sizes are required to detect associations under more genetically restrictive models. This interpretation is supported by genetic epidemiological simulation studies, which indicate that in complex diseases, the allele-comparison model typically provides greater statistical power for detecting variants with smaller effect sizes (Lee, 2015). Future studies should report results across multiple genetic models to facilitate meta-analyses and avoid false-negative conclusions. Concurrently, allele-based models should be regarded as more sensitive indicators for detecting weak-effect variants such as *MTHFR* C677T.

A key contribution of this study lies in confirming that the association between the *MTHFR* C677T genotype and dementia risk is consistent across racial groups. Through subgroup analyses of East Asian, European, South Asian, African/Mixed, and Middle Eastern populations, we found no significant heterogeneity in effect size across these groups. This finding contrasts with earlier concerns raised by population genetic studies. Pepe et al. (1998) and Wilcken et al. (2003) documented significant global differences in C677T allele frequency, prompting speculation that its effect on disease risk might vary by ethnicity. However, our results suggest that allele frequency differences do not translate into differential effect sizes. This is biologically plausible: if the C677T variant exerts its effect through a fundamental biochemical mechanism (reduced enzyme activity and elevated homocysteine), this mechanism should operate similarly across human populations, provided environmental conditions (such as folate intake) are comparable.

Under any genetic model, no significant association was found between the *MTHFR* A1298C polymorphism and dementia risk. This finding aligns with the majority of individual studies, including that of Mansoori et al. (2012), but contrasts with Coppedè et al. (2012), who proposed that compound heterozygous status (C677T/A1298C) may exert functional effects even with weak single-locus variants. From a biological perspective, the absence of an association observed is plausible. Compared to the C677T variant, which directly impacts enzyme thermal stability and reduces enzyme activity by 40–50% in TT homozygotes (Frosst et al., 1995), the A1298C variant exerts a relatively minor effect on enzyme function (Rozen, 1997). Furthermore, in the absence of the C677T variant, A1298C does not consistently elevate homocysteine levels, suggesting it may not be an independent risk factor for hyperhomocysteinemia-related pathologies. Nevertheless, we must acknowledge the potential for gene-gene interactions. Constrained by the limited number of studies reporting A1298C data, our analysis could not fully assess such interactive effects. Future studies with larger sample sizes and haplotype-based analyses are required to definitively exclude the role of A1298C in dementia susceptibility and to investigate whether its effects are contingent upon specific environmental conditions, such as extremely low folate levels.

This study has several limitations. The majority of included studies employed case-control designs, which have limited capacity for causal inference; sample sizes in certain racial subgroups were small, potentially affecting statistical power; and we were unable to control for confounding environmental factors such as folate and vitamin B12 levels, which are known to modulate the biochemical effects of *MTHFR* polymorphisms (Devlin et al., 2006; Kluijtmans et al., 2003); and only English-language literature was included. Although most analyses showed no statistical heterogeneity (*I*^2^ = 0%), this may reflect the limited number of studies and consistent reporting rather than true between-study homogeneity. Sensitivity analyses using random effects models confirmed the robustness of our findings. The null result for VaD should not be interpreted as evidence of no effect, as limited power may have obscured a modest association; therefore, the role of C677T in VaD warrants further investigation. Nevertheless, this study rigorously adhered to the PRISMA guidelines with a systematic and transparent literature screening process, innovatively incorporated dementia subtypes, multiple genetic models, and diverse ethnic populations into a stratified analysis framework, and demonstrated high overall methodological quality with no significant publication bias, all of which enhance the reliability of the findings.

Future work requires validation of current findings in larger, multicenter prospective cohorts, with a particular emphasis on strengthening research into VaD and underrepresented ethnic groups. Utilizing multi-omics technologies (such as epigenomics and metabolomics) to elucidate the specific downstream molecular pathways through which C677T influences AD pathology is crucial. Concurrently, conducting studies on gene-environment interactions will aid in revealing more precise risk patterns.

## 5 Conclusion

The *MTHFR* C677T polymorphism, particularly the T allele, is significantly associated with an increased risk of dementia, with this association primarily driven by AD. For VaD, while the effect estimate was in the same direction, no statistically significant association was observed in this meta-analysis. Under the other genetic models, no significant association was observed between the C677T polymorphism and dementia, suggesting that the allelic model may be a more sensitive indicator for detecting its risk effects. Subgroup analyses by ethnicity revealed no significant differences in the association between the C677T polymorphism and dementia. The *MTHFR* A1298C polymorphism did not show a significant association with the risk of AD or VaD under any genetic model.

In summary, *MTHFR* C677T represents a potential genetic risk marker for AD, whereas A1298C may not play a major role in this risk pathway. These findings generate the hypothesis that C677T genotyping might contribute to AD risk assessment in research contexts, but confirmation in large prospective cohorts and formal evaluation of its predictive utility are required before any translational application can be considered.

## Data Availability

The original contributions presented in the study are included in the article/Supplementary material, further inquiries can be directed to the corresponding authors.
