# Supplementary material for: MTHFR C677T and A1298C polymorphisms in dementia susceptibility: evidence from a meta-analytic investigation with disease-specific and ethnicity-stratified analyses
Source: Front Aging Neurosci. 2026 Aug 12;18:1858837. doi: 10.3389/fnagi.2026.1858837 (PMC13506712; doi:10.3389/fnagi.2026.1858837)
Supplement: Supplementary file 1 [file Data_Sheet_1.docx]

**Supplementary Table 1** The distributions of *MTHFR* C677T genotypes and allele numbers in dementia cases and controls

| Study | Genotype | | | | | | Alleles | | | |
| --- | --- | --- | --- | --- | --- | --- | --- | --- | --- | --- |
|  | CC | | CT | | TT | | C | | T | |
|  | Case | Control | Case | Control | Case | Control | Case | Control | Case | Control |
| **AD** | | | | | | | | | | |
| Luciano Belcavello,2014 | 26 | 70 | 52 | 78 | 4 | 13 | 104 | 218 | 60 | 104 |
| Xiu-Hua Bi,2009 | 82 | 90 | 179 | 172 | 125 | 113 | 343 | 352 | 429 | 398 |
| Mohammad Reza Keikhaee,2006 | 57 | 84 | 44 | 33 | 16 | 8 | 158 | 201 | 76 | 49 |
| Zahra Salimian Rizi,2024 | 52 | 46 | 33 | 48 | 3 | 6 | 137 | 140 | 39 | 60 |
| Midori Nishiyama,2000 | 15 | 13 | 36 | 15 | 16 | 5 | 66 | 41 | 68 | 25 |
| H. Wehr,2006 | 156 | 99 | 65 | 40 | 8 | 5 | 377 | 238 | 81 | 50 |
| D. Religa,2003 | 53 | 55 | 38 | 38 | 8 | 7 | 144 | 148 | 54 | 52 |
| Keshen Li,2009 | 37 | 69 | 92 | 103 | 69 | 68 | 166 | 241 | 230 | 239 |
| Neelam Chhillar,2014 | 31 | 69 | 58 | 26 | 11 | 5 | 120 | 164 | 80 | 36 |
| Liana Lisboa Fernandez,2005 | 10 | 15 | 17 | 11 | 2 | 3 | 39 | 41 | 21 | 17 |
| Davide Seripa,2003 | 49 | 45 | 63 | 43 | 12 | 9 | 161 | 133 | 87 | 61 |
| Davide Seripa,2003 | 30 | 28 | 67 | 55 | 29 | 23 | 127 | 111 | 125 | 101 |
| Khadidja Bouguerra,2024 | 27 | 42 | 49 | 43 | 30 | 19 | 103 | 127 | 109 | 81 |
| Nasim Mansoori,2012 | 51 | 89 | 26 | 29 | 3 | 2 | 128 | 207 | 32 | 33 |
| Nasir Abdelrafic Hamad,2024 | 18 | 57 | 20 | 9 | 35 | 7 | 56 | 123 | 90 | 23 |
| Nasser Attia Elhawary,2013 | 18 | 20 | 17 | 10 | 8 | 2 | 53 | 50 | 33 | 14 |
| Rivka Dresner Pollak,2000 | 30 | 29 | 49 | 37 | 13 | 16 | 109 | 95 | 75 | 69 |
| Vanessa Cavalcante da Silva, 2006 | 19 | 25 | 19 | 23 | 5 | 2 | 57 | 73 | 29 | 27 |
| Maria Styczynska, 2008 | 79 | 97 | 59 | 67 | 17 | 12 | 217 | 261 | 93 | 91 |
| Leila Mansouri, 2013 | 13 | 52 | 24 | 39 | 1 | 9 | 50 | 143 | 26 | 57 |
| Yaling Jiang, 2021 | 337 | 188 | 340 | 168 | 44 | 9 | 1014 | 544 | 428 | 186 |
| Stephen P. McIlroy, 2002 | 59 | 50 | 20 | 19 | 4 | 2 | 138 | 119 | 28 | 23 |
| **VaD** | | | | | | | | | | |
| Pengpeng Jin,2013 | 132 | 152 | 99 | 96 | 73 | 52 | 363 | 400 | 245 | 200 |
| Gurjinder Kaur,2018 | 162 | 179 | 55 | 86 | 5 | 11 | 379 | 444 | 65 | 108 |
| Zuliani G,2001a | 20 | 17 | 26 | 25 | 14 | 12 | 66 | 59 | 54 | 49 |
| Midori Nishiyama,2000 | 9 | 13 | 17 | 15 | 9 | 5 | 35 | 41 | 35 | 25 |
| Lei Chen,2023 | 30 | 40 | 86 | 97 | 75 | 45 | 146 | 177 | 236 | 187 |
| Nasim Mansoori,2012 | 35 | 89 | 14 | 29 | 1 | 2 | 84 | 207 | 16 | 33 |
| Rivka Dresner Pollak,2000 | 34 | 29 | 41 | 37 | 10 | 16 | 109 | 95 | 61 | 69 |
| G. Zuliani, 2001b | 17 | 11 | 34 | 8 | 17 | 1 | 68 | 30 | 68 | 10 |
| Stephen P. McIlroy, 2002 | 31 | 50 | 37 | 19 | 8 | 2 | 99 | 119 | 53 | 23 |

**Supplementary Table 2** The distributions of *MTHFR* A1298C genotypes and allele numbers in dementia cases and controls

| Study | Genotype | | | | | | Alleles | | | |
| --- | --- | --- | --- | --- | --- | --- | --- | --- | --- | --- |
|  | AA | | AC | | CC | | A | | C | |
|  | Case | Control | Case | Control | Case | Control | Case | Control | Case | Control |
| **AD** | | | | | | | | | | |
| Khadidja Bouguerra,2024 | 54 | 25 | 37 | 21 | 11 | 5 | 145 | 71 | 59 | 31 |
| Nasim Mansoori,2012 | 20 | 44 | 41 | 59 | 19 | 17 | 81 | 147 | 79 | 93 |
| Nasir Abdelrafic Hamad,2024 | 28 | 26 | 20 | 24 | 25 | 23 | 76 | 76 | 70 | 70 |
| Vanessa Cavalcante da Silva, 2006 | 1 | 1 | 21 | 22 | 21 | 27 | 23 | 24 | 63 | 76 |
| Leila Mansouri, 2013 | 0 | 0 | 23 | 7 | 15 | 93 | 23 | 7 | 53 | 193 |
| Yaling Jiang, 2021 | 464 | 240 | 240 | 122 | 17 | 0 | 1168 | 608 | 274 | 122 |
| **VaD** | | | | | | | | | | |
| Lei Chen,2023 | 148 | 122 | 46 | 57 | 2 | 3 | 342 | 301 | 50 | 63 |
| Nasim Mansoori,2012 | 17 | 44 | 27 | 59 | 6 | 17 | 61 | 147 | 39 | 93 |

**Supplementary Table 3** Quality assessment of NOS

| study | Selection | Comparability | Outcome | Total |
| --- | --- | --- | --- | --- |
| Pengpeng Jin,2013 | 4 | 1 | 2 | 7 |
| Luciano Belcavello,2014 | 3 | 2 | 2 | 7 |
| Xiu-Hua Bi,2009 | 4 | 2 | 2 | 8 |
| Gurjinder Kaur,2018 | 3 | 2 | 3 | 8 |
| Mohammad Reza Keikhaee,2006 | 3 | 2 | 2 | 7 |
| Zuliani G,2001a | 3 | 2 | 2 | 7 |
| Zahra Salimian Rizi,2024 | 3 | 2 | 2 | 7 |
| Midori Nishiyama,2000 | 3 | 1 | 2 | 6 |
| H. Wehr,2006 | 3 | 2 | 2 | 7 |
| D. Religa,2003 | 3 | 2 | 3 | 8 |
| Keshen Li,2009 | 4 | 2 | 3 | 9 |
| Neelam Chhillar,2014 | 3 | 2 | 2 | 7 |
| Liana Lisboa Fernandez,2005 | 3 | 2 | 2 | 7 |
| Davide Seripa,2023 | 4 | 2 | 3 | 9 |
| Khadidja Bouguerra,2024 | 3 | 2 | 2 | 7 |
| Lei Chen,2023 | 3 | 2 | 3 | 8 |
| Nasim Mansoori,2012 | 4 | 1 | 3 | 8 |
| Nasir Abdelrafic Hamad,2024 | 4 | 1 | 3 | 8 |
| Nasser Attia Elhawary,2013 | 3 | 1 | 2 | 6 |
| Rivka Dresner Pollak,2000 | 4 | 2 | 1 | 7 |
| Vanessa Cavalcante da Silva, 2006 | 3 | 2 | 2 | 7 |
| Maria Styczynska, 2008 | 3 | 1 | 2 | 6 |
| Leila Mansouri, 2013 | 3 | 2 | 2 | 7 |
| G. Zuliani, 2001b | 4 | 2 | 2 | 8 |
| Yaling Jiang, 2021 | 4 | 2 | 2 | 8 |
| Stephen P. McIlroy, 2002 | 4 | 2 | 2 | 8 |

**Supplementary Table 4** Quality assessment of Q-Genie

| study | 1 | 2 | 3 | 4 | 5 | 6 | 7 | 8 | 9 | 10 | 11 | Total |
| --- | --- | --- | --- | --- | --- | --- | --- | --- | --- | --- | --- | --- |
| Pengpeng Jin,2013 | 6 | 5 | 5 | 6 | 6 | 7 | 5 | 6 | 4 | 3 | 5 | 58 |
| Luciano Belcavello,2014 | 6 | 4 | 3 | 5 | 5 | 7 | 6 | 3 | 4 | 3 | 5 | 51 |
| Xiu-Hua Bi,2009 | 6 | 5 | 6 | 6 | 6 | 7 | 6 | 6 | 4 | 3 | 5 | 60 |
| Gurjinder Kaur,2018 | 6 | 5 | 7 | 5 | 5 | 6 | 6 | 3 | 4 | 3 | 5 | 55 |
| Mohammad Reza Keikhaee,2006 | 6 | 6 | 4 | 5 | 7 | 7 | 6 | 3 | 3 | 3 | 5 | 55 |
| Zuliani G,2001a | 6 | 4 | 3 | 4 | 3 | 7 | 4 | 3 | 3 | 3 | 5 | 45 |
| Zahra Salimian Rizi,2024 | 6 | 6 | 4 | 6 | 7 | 7 | 6 | 6 | 3 | 3 | 5 | 59 |
| Midori Nishiyama,2000 | 6 | 3 | 3 | 5 | 4 | 6 | 4 | 4 | 3 | 3 | 4 | 45 |
| H. Wehr,2006 | 6 | 5 | 5 | 5 | 4 | 7 | 5 | 5 | 3 | 3 | 5 | 53 |
| D. Religa,2003 | 6 | 5 | 6 | 5 | 4 | 7 | 6 | 6 | 3 | 3 | 5 | 56 |
| Keshen Li,2009 | 6 | 5 | 5 | 5 | 6 | 6 | 4 | 7 | 3 | 3 | 5 | 55 |
| Neelam Chhillar,2014 | 6 | 5 | 4 | 5 | 3 | 7 | 5 | 4 | 3 | 3 | 5 | 50 |
| Liana Lisboa Fernandez,2005 | 6 | 5 | 3 | 5 | 4 | 7 | 5 | 5 | 3 | 3 | 5 | 51 |
| Davide Seripa,2023 | 7 | 7 | 7 | 6 | 7 | 7 | 6 | 5 | 4 | 7 | 6 | 69 |
| Khadidja Bouguerra,2024 | 6 | 5 | 5 | 5 | 6 | 6 | 5 | 4 | 3 | 3 | 5 | 53 |
| Lei Chen,2023 | 7 | 5 | 5 | 5 | 4 | 6 | 7 | 3 | 3 | 3 | 5 | 53 |
| Nasim Mansoori,2012 | 6 | 5 | 4 | 6 | 5 | 7 | 6 | 7 | 3 | 3 | 5 | 57 |
| Nasir Abdelrafic Hamad,2024 | 6 | 5 | 4 | 5 | 4 | 6 | 5 | 3 | 3 | 3 | 5 | 49 |
| Nasser Attia Elhawary,2013 | 6 | 5 | 4 | 5 | 6 | 7 | 5 | 3 | 3 | 3 | 5 | 52 |
| Rivka Dresner Pollak,2000 | 6 | 7 | 6 | 5 | 6 | 6 | 6 | 3 | 3 | 3 | 6 | 57 |
| Vanessa Cavalcante da Silva, 2006 | 6 | 5 | 4 | 5 | 6 | 7 | 5 | 3 | 3 | 3 | 5 | 52 |
| Maria Styczynska, 2008 | 6 | 5 | 6 | 5 | 5 | 7 | 6 | 5 | 3 | 3 | 5 | 56 |
| Leila Mansouri, 2013 | 6 | 5 | 3 | 5 | 4 | 7 | 5 | 3 | 3 | 3 | 5 | 49 |
| G. Zuliani, 2001b | 6 | 5 | 3 | 5 | 4 | 7 | 5 | 3 | 3 | 3 | 5 | 49 |
| Yaling Jiang, 2021 | 7 | 5 | 7 | 6 | 6 | 7 | 6 | 6 | 6 | 3 | 6 | 65 |
| Stephen P. McIlroy, 2002 | 6 | 7 | 5 | 5 | 5 | 7 | 7 | 4 | 3 | 3 | 6 | 58 |

**Supplementary Methods:detailed search strategy in four databases.**

**1Pubmed**

| Search | Query | Results |
| --- | --- | --- |
| #1 | **((((((("Methylenetetrahydrofolate Reductase (NADPH2)"[Mesh]) OR (methylenetetrahydrofolate reductase[Title/Abstract])) OR (MTHFR[Title/Abstract])) OR (C677T[Title/Abstract])) OR (A1298C[Title/Abstract])) OR (rs1801133[Title/Abstract])) OR (rs1801131[Title/Abstract])) OR (C.665C>T[Title/Abstract])** | 9675 |
| #2 | **((((("Polymorphism, Genetic"[Mesh]) OR (polymorphism[Title/Abstract])) OR (variant[Title/Abstract])) OR (mutation[Title/Abstract])) OR (genotype[Title/Abstract])) OR (genetic association[Title/Abstract])** | 1,202,070 |
| #3 | (("Alzheimer Disease"[Mesh]) OR (Alzheimer's disease[Title/Abstract])) OR (AD[Title/Abstract]) | 340,042 |
| #4 | (("Dementia, Vascular"[Mesh]) OR (vascular dementia[Title/Abstract])) OR (VaD[Title/Abstract]) | 22,255 |
| #5 | #3 OR #4 | 354,821 |
| #6 | #1 AND #2 AND #5 | 142 |

**2Cochrane**

| Search | Query | Results |
| --- | --- | --- |
| #1 | MeSH descriptor: [Methylenetetrahydrofolate Reductase (NADPH2)] explode all trees | 191 |
| #2 | (MTHFR):ti,ab,kw OR (methylenetetrahydrofolate reductase):ti,ab,kw OR (C677T):ti,ab,kw OR (A1298C):ti,ab,kw OR (rs1801133):ti,ab,kw OR (rs1801131):ti,ab,kw OR (C.665C>T):ti,ab,kw | 448 |
| #3 | #1 OR #2 | 448 |
| #4 | MeSH descriptor: [Polymorphism, Genetic] explode all trees | 4856 |
| #5 | (polymorphism):ti,ab,kw OR (variant):ti,ab,kw OR (mutation):ti,ab,kw OR (genotype):ti,ab,kw OR (genetic association):ti,ab,kw | 39173 |
| #6 | #4 OR #5 | 39210 |
| #7 | MeSH descriptor: [Alzheimer Disease] explode all trees | 6116 |
| #8 | (Alzheimer's disease):ti,ab,kw OR (AD):ti,ab,kw | 35352 |
| #9 | #7 OR #8 | 35352 |
| #10 | MeSH descriptor: [Dementia, Vascular] explode all trees | 473 |
| #11 | (vascular dementia):ti,ab,kw OR (VaD):ti,ab,kw | 2508 |
| #12 | #10 OR #11 | 2574 |
| #13 | #9 OR #12 | 37039 |
| #14 | #3 AND #6 AND #13 | 4 |

**3Embase**

| Search | Query | Results |
| --- | --- | --- |
| #1 | 'methylenetetrahydrofolate reductase (nadph2)'/exp OR 'methylenetetrahydrofolate reductase (nadph2)' OR 'mthfr':ab,ti OR 'c677t':ab,ti OR 'a1298c':ab,ti OR 'rs1801133':ab,ti OR 'rs1801131':ab,ti OR 'c.665c>t':ab,ti | 12,955 |
| #2 | 'genetic polymorphism'/exp OR 'genetic polymorphism' OR 'polymorphism':ab,ti OR 'variant':ab,ti OR 'mutation':ab,ti OR 'genotype':ab,ti OR 'genetic association':ab,ti | 1,730,536 |
| #3 | 'alzheimer disease'/exp OR 'alzheimer disease' OR 'ad':ab,ti | 510,307 |
| #4 | 'multiinfarct dementia'/exp OR 'multiinfarct dementia' OR 'vascular':ab,ti AND 'dementia':ab,ti OR 'vad':ab,ti | 44,783 |
| #5 | #7 OR #8 | 538,623 |
| #6 | #1 AND #2 AND #5 | 232 |

**4WOS**

| Search | Query | Results |
| --- | --- | --- |
| #1 | ((((((TS=(methylenetetrahydrofolate reductase)) OR TS=(MTHFR)) OR TS=(C677T)) OR TS=(A1298C)) OR TS=(rs1801133)) OR TS=(rs1801131)) OR TS=(C.665C>T) | 13,553 |
| #2 | **(((((TS=(Polymorphism, Genetic)) OR TS=(polymorphism)) OR TS=(variant)) OR TS=(mutation)) OR TS=(genotype)) OR TS=(genetic association)** | 2,559,907 |
| #3 | (((((TS=(Alzheimer Disease)) OR TS=(AD)) OR TS=(Alzheimer's disease)) OR TS=(Dementia, Vascular)) OR TS=(vascular dementia)) OR TS=(VaD) | 549,645 |
| #4 | #1 AND #2 AND #3 | 290 |

**Supplementary Figure 1.** Forest plot of the *MTHFR* C677T polymorphism with fixed effects model. a. homozygote model(TT vs CC) b. heterozygote model( CT vs CC) c. dominant model( TT+CT vs CC) d. recessive model(TT vs CC+CT)

**Supplementary Figure 2.** Forest plots for the association between *MTHFR* C677T polymorphism and AD/VaD with fixed effect model. a. homozygote model(TT vs CC) b. heterozygote model( CT vs CC) c. dominant model( TT+CT vs CC) d. recessive model(TT vs CC+CT)

**Supplementary Figure 3.** Forest plots for the association between *MTHFR* C677T polymorphism and different ethnicity with fixed effect model. a. allele contrast model(T vs C) b. homozygote model(TT vs CC) c. heterozygote model( CT vs CC) d. dominant model( TT+CT vs CC) e. recessive model(TT vs CC+CT)

**Supplementary Figure 4.** Forest plot of the *MTHFR* A1298C polymorphism with fixed effects model. a. allele contrast model(C vs A) b. homozygote model(CC vs AA) c. heterozygote model( AC vs AA) d. dominant model(AC + CC vs AA) e. recessive model(CC vs AA+AC)

**Supplementary Figure 5.** Funnel plots. a. allele contrast model(T vs C) b. homozygote model(TT vs CC) c. heterozygote model( CT vs CC) d. dominant model( TT+CT vs CC) e. recessive model(TT vs CC+CT)
